# Operationalizing soil persistence in soil and ecosystem ecology

**DOI:** 10.1111/nph.71492

**Published:** 2026-08-02

**Authors:** Matthias C. Rillig, Laurent Philippot, Johannes Lehmann

**Affiliations:** ^1^ Freie Universität Berlin Institut für Biologie 14195 Berlin Germany; ^2^ Université de Bourgogne Franche‐Comté INRAE, Institute Agro Dijon, Agroecologie 21000 Dijon France; ^3^ School of Integrative Plant Science Cornell University Ithaca NY 14853 USA; ^4^ Cornell Atkinson Center for Sustainability Cornell University Ithaca NY 14853 USA

**Keywords:** erosion, It's The Song Not The Singer Theory, plant–soil interactions, resilience, soil health, system collapse, terrestrial ecosystems

## Abstract

Trade‐offs between soil persistence and the provision of certain soil services and activity/functions.

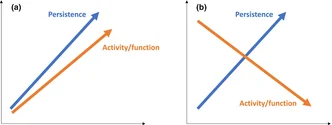

## Disclaimer

The New Phytologist Foundation remains neutral with regard to jurisdictional claims in maps and in any institutional affiliations.

## Introduction

Soils underpin the majority of carbon and nutrient cycling processes in terrestrial ecosystems (Chapin *et al*., 2011), and are crucial for food security (Oliver & Gregory, 2015) and One Health (Banerjee & van der Heijden, 2023), and they harbor most of the planet's biodiversity (Anthony *et al*., 2023). Given this enormous importance, it is understandable that much of soil ecology has focused on understanding the delivery of these critical services. This paper argues for a shift in approach, especially in terms of studying plant–soil interactions: a ‘soils‐as‐equal’ to plants perspective. As previously suggested (Strullu‐Derrien & Rillig, 2026), soil can be viewed not merely from the vantage point of supporting plant growth (including crop yield), a view common in much work on soil, plant–soil interactions or ecosystem ecology in general (van Breemen, 1993; Chapin *et al*., 2011). By inverting the traditional view centered on aspects of primary production, we can examine soil through the lens of persistence, the capacity of a soil to continue to exist across time. Persistence is generally defined as the time between when a system arises and when it collapses (Cumming & Peterson, 2017; Lenton *et al*., 2021). Collapse and system identity (what characterizes the entity that collapses?) are tricky to define (Cumming & Peterson, 2017), and will need to be operationalized for a given study.

To practically facilitate this shift in perspective, it is helpful to define the individual soil. A scale‐invariant definition appears most appropriate: soil is the confluence of weathering minerals, atmosphere, water, organic matter (specifically microbially derived organic matter), and living biota. By decoupling soil from macroscopic features, such as horizons and vegetation, we recognize that a soil can exist at much smaller scales than traditionally considered, provided the requisite confluence of components is present.

We posit that soil, as a complex system defined above, exhibits a trajectory toward persistence. This framing is a non‐normative, heuristic device, inspired by the proposal of the ‘It's the Song, Not the Singer’ (ITSNTS) theory of evolution (Doolittle & Inkpen, 2018). Here, the soil is the ‘song’ or rather the ‘symphony’ as a continuous biogeochemical performance to which a myriad of soil biota (the singers) contribute in shifting composition through time (Falkowski *et al*., 2008). While this view cannot be falsified (Doolittle & Inkpen, 2018), it provides a useful framework for generating new research questions regarding system persistence (sometimes also referred to as system survival) (Lenton *et al*., 2021), the central tenet of ITSNTS theory.

Soil persistence is distinct from resilience of soils, but related. Resilience describes the ability of a soil to return to a baseline state of biological function or community composition following a disturbance (Seybold *et al*., 1999); this concept is focused on speed of recovery, the faster the greater the resilience, and we argue that the resilience concept is thus more focused on the ‘singers’. Persistence is focused on the ‘song’, on the uninterrupted longer‐term existence of the soil entity. A soil function or community can be resilient, but the soil can still be lost (i.e. not persist) in the long term if erosion rates are high. But the two concepts are also related: less resilient systems make a collapse (end of persistence) more likely (Cumming & Peterson, 2017).

Such theoretical considerations aside, persistence is highly relevant from a more applied perspective, such as in the contexts of soil health (Lehmann *et al*., 2020) and in agricultural sustainability, especially against the backdrop of high soil loss rates globally (Borrelli *et al*., 2017). Soil health indicators often maximize process rates, whereas soil persistence depends on minimizing net losses and maintaining system integrity over time. Clearly, thinking about persistence of soils is therefore important, and, in general terms, all efforts to enhance the sustainability of soils also favor properties important for persistence. Yet, persistence of soils plays almost no role in current experimental soil ecology. This is in part because the concept is not operationalized for use in experiments, and persistence seems at first sight no different from studying soil functions following the *status quo*. In the following sections, we propose an operational way to include soil persistence attributes in measurement protocols and questions/hypotheses in soil ecological experiments, and we point out specific cases where the interpretation of measurements of soil persistence and of soil functions may be at odds.

### Operationalizing persistence in experimental settings

While soil persistence is traditionally explored through observational data (covering time frames of decades to centuries; Evans *et al*., 2020), we propose that persistence can be operationalized as a set of response variables to consider in many laboratory experiments.

The primary and most absolute mode of soil loss is via erosion. Therefore, any measures of structural integrity, such as soil aggregate stability, will serve as critical proxies for persistence (Kemper & Rosenau, 2018). Experimental readouts should focus not only on macro‐ and micro‐aggregate stability, mechanisms underpinning soil stability, such as the production of biogenic soil aggregate binding agents (e.g. extracellular polymeric substances) but also on the direct quantification of erosion rates within microcosms or mesocosms, a parameter currently heavily underrepresented in soil microbial ecology (Philippot *et al*., 2024).

Beyond physical loss through erosion or radical shift in habitat, we could also consider indicators of functional persistence. A soil that experiences a collapse in biodiversity or a significant loss in key process rates (Rillig *et al*., 2019) may be considered on a ‘loss trajectory’. For such analyses, time course data would be key, since they permit analysis of the slope of functional change over time to predict the point of failure.

The persistence of a soil entity is not merely a function of its resistance to loss, but can be defined by the dynamic equilibrium between erosion and pedogenesis. Soil formation processes are thus an important part of the equation of soil persistence overall. To more fully operationalize this soil persistence framework, we must therefore also use experimental indicators for soil formation, such as mineral weathering (Philippot *et al*., 2024) and input of organic matter (Gregory, 2022). Measuring the flux of cations from primary minerals or the formation of secondary minerals are parameters to consider; at the micro‐scale, one could measure the relative ability of soil microbes to colonize new volumes of parent material (Echenique‐Subiabre *et al*., 2025) and cause the transformation of soil minerals. Rarely are these parameters currently assayed in soil ecology experiments or studies on soil health. Going forward, soil ecologists can routinely use ‘parent material bags’ in addition to litter bags in their experiments to measure parameters indicative of soil formation, such as the accrual of microbially derived carbon.

A different experimental framework, that of matter‐closed but energy‐open systems can also be very useful for the study of soil persistence. Miniature versions of such matter‐closed systems (microbial biospheres; Rillig & Antonovics, 2019) could be filled with a soil‐like substrate to observe patterns of system persistence; currently, almost all such experimental systems are aquatic (Li *et al*., 2026). Experimental innovations will be necessary to study soil erosion in such microbial biospheres, but the cessation of biogeochemical cycles over time, such as the carbon cycle, could be readily captured in time‐series measurements.

### Shifting questions and soil persistence‐function trade‐offs

Focusing on soil persistence can offer new questions for experiments, flipping around more well‐rehearsed approaches. For example, rather than asking which soil properties are best suited for plants, one can ask which plant traits, species, or plant assemblages are most supportive of soil persistence; an approach with obvious applications in soil restoration (Neuenkamp *et al*., 2024). Rather than asking how soil pollutants or other factors of global change impair soil functions (Rillig *et al*., 2019), about which there is comparatively much known, we could ask how they challenge soil persistence. Perhaps soil chronosequences or comparisons of geologically old and relatively young soils, or even topsoil/subsoil comparisons, can be leveraged to learn about soil persistence mechanisms in operation over longer time scales.

Perhaps the most significant implication of the soil persistence framework is the potential conflict between ‘soil functioning’ (as currently defined mostly with respect to delivering services to plants and ecosystems) and soil persistence. For example, in the current view, high rates of decomposition and mineralization (and thus also soil respiration) are viewed as ‘positive’ functions, because this process delivers plant‐available nutrients, and respiration is frequently used as a general indicator of soil biological activity. However, from the perspective of the soil entity, rapid decomposition represents the loss of the organic binding agents necessary for stabilizing soil aggregates (Tisdall & Oades, 1982; Six *et al*., 2004), and thus extremely efficient decomposition may not be positive for soil persistence. The same argument also holds for all soil activity measures that are related to decomposition, such as enzymatic activity related to carbon, nitrogen or phosphorus processing. Perhaps another such conflict exists with respect to the interpretation of soil water repellency, which from an agronomic/urban ecosystem perspective is undesirable, because it limits water infiltration and favors surface run‐off. However, for the soil, a certain degree of water repellency may be positive for persistence because of positive correlations with soil aggregate stability (Zheng *et al*., 2016). High hydrological connectivity within soils can enhance water flow and facilitate the transport of dissolved nutrients (Vereecken *et al*., 2022). However, from a persistence perspective, increased connectivity can also amplify the physical export of soil material as enhanced water flow pathways promote the detachment and transport of fine particles. Even root growth and rhizodeposition could offer such a point of potential ‘conflict’ to be explored, because the priming effect (Fontaine *et al*., 2007), caused by the stimulation of microbial activity by the provision of easily metabolizable plant‐derived substances, could lead to soil organic matter losses. Perhaps excessive nutrient mining, by microbes, fungal root symbionts or roots themselves, could be construed as overexploitation of the soil's nutrient reserves, even though this process is arguably highly desirable from the perspective of primary production. Activities of soil fauna such as earthworms and termites play a role in soil health by promoting mixing, aeration, and nutrient cycling (Lejoly *et al*., 2026). These activities can stimulate microbial processes and improve resource availability for plants and other organisms (Wu *et al*., 2025). However, from a persistence perspective, bioturbation can be negative by disrupting soil aggregates and weakening structural integrity: the breakdown of aggregates exposes previously protected organic matter, thereby accelerating decomposition.

In several cases, benefits for soil functions/services and persistence may be well aligned (Fig. 1a), for example supplying nutrients to benefit plant cover, or mycorrhizal fungal activity benefiting plant growth and stabilizing soil. In all cases of potential trade‐offs (Fig. 1b), it is a nuanced question of process rates; that is rates of nutrient mining and decomposition. For a given soil, from what point on do the benefits of a process rate (like decomposition) or management intervention for plant growth and other ecosystem processes outweigh the negative effects on persistence? And how do different soils differ in this respect? How can we minimize these trade‐offs? This is an important set of new research questions.

**Fig. 1:**
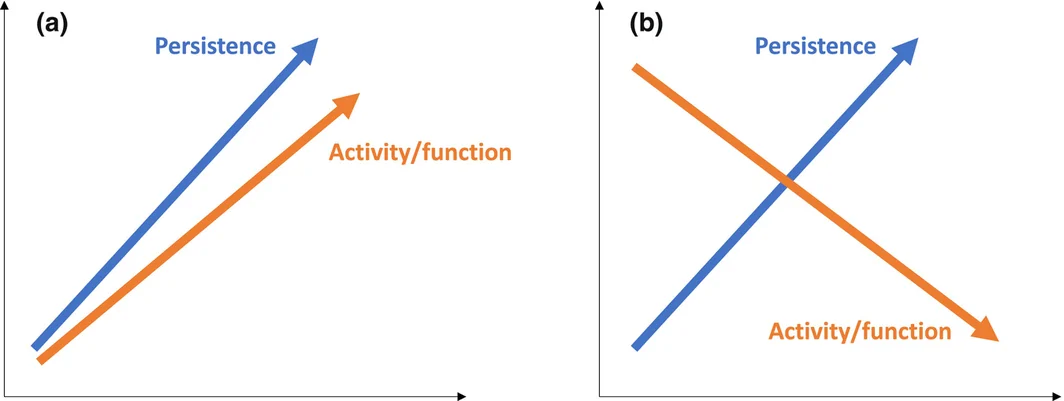
Trade‐offs between soil persistence and the provision of certain soil services and activity/functions. With increasing process rates (*x*‐axis, such as decomposition or nutrient mining from minerals; this could also stand for management interventions), certain soil functions will be maximized, for example those related to favoring primary production. In some cases (a), soil persistence will be well aligned with these functions; that is, what is good for the soil function will also be good for persistence. But there are also cases (b) where soil persistence and soil functions are at odds: if a process increases soil functions, it may be deleterious for soil persistence. An important set of research questions pertain to this balance between the soil services and soil persistence.

## Conclusions

Systematically exploring the perspective of soil persistence in hypothesis‐driven experiments in plant–soil ecology will likely uncover new insights into soil ecology, but also into terrestrial ecosystems as a whole. We should reframe soil health more decisively in terms of persistence: a healthy soil is one that will best persist when faced with various external drivers, rather than one that will deliver more agronomically/urban‐system relevant functions. Thus, soil health indicators should be complemented by persistence metrics to avoid systematically favoring high‐activity but low‐stability systems. Soils globally are at risk of being lost, a pattern exacerbated by unfolding global change, including climate change, land use change, and pollution. An interdisciplinary, experiment‐driven research effort aimed at soil persistence seems in order. Additionally, a view of soils as ancient, persistent systems might also help in communicating the value of soils to broader audiences (Trdlicova & Neilson, 2025).

## Competing interests

None declared.

## Author contributions

MCR wrote the first draft of the manuscript. LP and JL contributed ideas and helped finalize the manuscript.
